# Upregulation of KIF20A promotes tumor proliferation and invasion in renal clear cell carcinoma and is associated with adverse clinical outcome

**DOI:** 10.18632/aging.202153

**Published:** 2020-11-24

**Authors:** Xiaohan Ren, Xinglin Chen, Yisheng Ji, Lin Li, Yunxin Li, Chao Qin, Kai Fang

**Affiliations:** 1 Department of Urology, Shanghai Pudong Hospital, Fudan University Pudong Medical Center, Huinan, Pudong 201399, Shanghai, China; 2 The State Key Lab of Reproductive, Department of Urology, The First Affiliated Hospital of Nanjing Medical University, Nanjing 210029, China; 3 The First Clinical Medical College, Nanjing Medical University, Nanjing 211166, P.R. China

**Keywords:** KIF20A, renal cell carcinoma

## Abstract

Extensive research has revealed the pivotal role of kinesin family member 20A (KIF20A) in cancer. However, its latent involvement in renal clear cell carcinoma (ccRCC) still remains unclear. Thus, here we explored the role of KIF20A in ccRCC. For this, a series of software including R (v. 3.6.1), SPSS (v. 23), ImageJ and FlowJo were used for the analyses. Open-access data were obtained from The Cancer Genome Atlas (TCGA), International Cancer Genome Consortium (ICGC) and Gene Expression Omnibus (GEO) databases. Weighted Gene Co-expression Network Analysis (WGCNA) was used for module gene identification. In vitro results indicated that KIF20A expression is up-regulated in ccRCC tissue. KIF20A knockdown was able to inhibite cell proliferation and invasion of kidney A498 and Caki-1 cells. Meanwhile, KIF20A showed a strong association with immune infiltration. Particularly, KIF20A had a strong positive correlation with Th2 cells, Treg cells and Macrophages, but a negative correlation with Th17 cells, Mast cells and NK cells. These correlations may suggest the use of KIF20A as an underlying immunotherapy target in ccRCC. Our data indicated that KIF20A may promote cell invasion and proliferation in ccRCC, thus serving as an independent tumor marker and a putative therapeutic target.

## INTRODUCTION

RCC is the prevalent kidney malignancy that largely arises from the renal tubular epithelium. It has been estimated that 300,000 new cases and 130,000 deaths occur due to RCC each year, possibly in response to changes in personal habits, lifestyle and increased life expectancy [1]. As a multifactor disease, the etiology of RCC is particularly impacted by a number of factors, such as obesity, smoking, hypertension (as well as antihypertensive treatment) and biological inheritance. So far, the three most frequent histological subtypes are clear cell RCC (ccRCC) (70% -80% of the cases), papillary RCC (10%-20% of the cases), and chromophobe RCC (5% of the cases) [2].

Despite the comparatively lower malignant degree, the incidence and mortality due to ccRCC have rapidly increasing worldwide, especially in low- and middle-income countries [3]. Since most of the ccRCC cases are insensitive to radiotherapy or chemotherapy, surgery is considered the first choice of treatment for early and locally advanced ccRCC. Still, approximately 30% of ccRCC patients present distant metastasis after treatment. Meanwhile, no effective treatment for terminal ccRCC has been defined [4]. In fact, since tumor cells haile from renal parenchyma, they can easily infiltrate into the renal capsule and further develop into hemangioma embolus or metastasis, making it more difficult to manage the disease progression [5]. Therefore, the discovery of useful biomarkers for early diagnosis and predicting the prognosis of ccRCC is highly warranted.

More recently, with increasing focus on the molecular level of ccRCC, a growing body of evidence has indicated that various genes (and related regulatory networks) are crucial for tumorigenesis and cell development [6]. KIF20A, also known as MKLP2 and RAB6KIFL, is located on the chromosome 5q31.2 and encodes a protein consisting of 890 amino acid residues [7]. As a member of the KIF family, KIF20A is mainly distributed in the central region of mitotic spindles and, as such, participated in the processes driving cell division (mitosis) [8]. A number of studies have recently shown a prominent increase on the level of KIF20A expression in varieties of malignancies, including breast, lung, liver, gastric cancers, thus indicating a putative role of KIF20A in cancer development and progression [9–11]. Moreover, KIF20A overexpression appears to be strongly associated with poor prognosis and clinical parameters of tumor patients. Based on immunohistochemistry (IHC) and in vitro analyses, Kawai and colleagues have found that KIF20A may significantly promote the proliferation of ovarian clear-cell carcinoma cells [12]. Xiong and colleagues have also reached some similar conclusions, in which KIF20A can promote the proliferation of colorectal cancer cells via the JAK/STAT3 signaling pathway [13]. Moreover, several studies have noted the underlying importance of KIF20A in immunotherapeutics. Upon performing some genome-wide cDNA microarray analysis in pancreatic cancer, Imai and colleagues have regarded KIF20A as a novel and promising target for anticancer immunotherapy. Their result have demonstrated that KIF20A-2, -8 and -28 might induce HLA-A2-restricted cytotoxic T lymphocytes in HLA-A2 Tgm [14]. Meanwhile, the vaccination with a KIF20A-derived SP may induce a KIF20A-specific CTL response and, therefore, yield promising results in patients affected by advanced cancer [15].

However, so far, no relevant literature has demonstrated the putative role of KIF20A in ccRCC. Therefore, here we aimed to investigate the role of KIF20A in ccRCC and thus provide new directions for future cancer research. Upon analysis of the TCGA database, here we first identified an elevation of KIF20A expression in tumors when compared with normal kidney tissue. Thereafter, we evaluated the association between the KIF20A expression and the clinical pathology, survival and immune cell content (or infiltration) of ccRCC by the bioinformatics analysis. In vitro experiments suggest that KIF20A is highly expressed in ccRCC tissues and possibly responsible by promoting tumor proliferation and invasion. Collectively, our data indicate that KIF20A may serve as a valuable therapeutic target for ccRCC treatment.

## RESULTS

### KIF20A expression profiling

**Figure 1:**
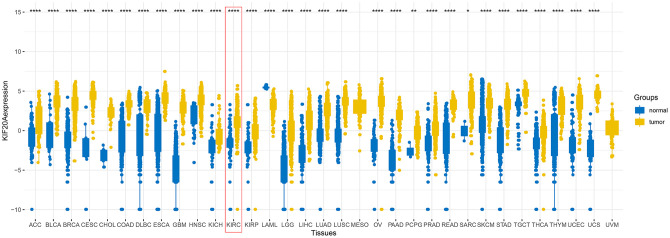
**The expression of KIF20A after merging TCGA and GTEx databases.**

**Figure 2:**
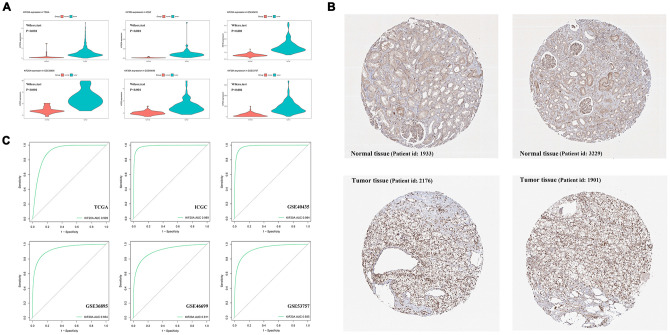
**The analysis of KIF20A expression profile.** (**A**) The mRNA expression of KIF20A is up-regulated in TCGA, ICGC, GSE40435, GSE36895, GSE46699 and GSE53757; (**B**) Representative IHC pictures from HPA database.; (**C**) ROC curve of KIF20A in predicting tumorigenesis. Abbreviations: TCGA: The Cancer Genome Atlas; ICGC: International Cancer Genome Consortium; IHC: immunohistochemistry; HPA: The Human Protein Atlas project.

Upon merging the scaled matrix of KIF20A expression from both TCGA and GTEx databases, we observed that KIF20 was highly expressed in a variety of cancer types, including KIRC (Figure 1). Increased levels of KIF20A expression were also observed in renal cancer, according to TCGA, ICGC and GSE (GSE40435, GSE36895, GSE46699 and GSE53757) databases (P<0.001) (Figure 2A). Representative immunohistochemical images illustrating KIF20A protein levels (obtained from the HPA database) are shown in Figure 2B. These results indicate some stronger staining intensity and quantity along the tumor tissue (Patient id: 1933, Cells in glomeruli: Staining = high, Intensity = strong, Quantity > 75%, Cells in tubules: Staining = high, Intensity = strong, Quantity = 25%-75%; Patient id: 3229, Cells in glomeruli: Staining = medium, Intensity = moderate, Quantity > 75%, Cells in tubules: Staining = medium, Intensity = moderate, Quantity > 75%; Patient id: 2176: Staining = high, Intensity = strong, Quantity > 75%; Patient id: 1901: Staining = high, Intensity = strong, Quantity > 75%). The ROC curve revealed that KIF20 had a compelling sensitivity and specificity to predict the occurrence of ccRCC (Figure 2C).

### Clinical correlation and prognosis analysis

**Figure 3:**
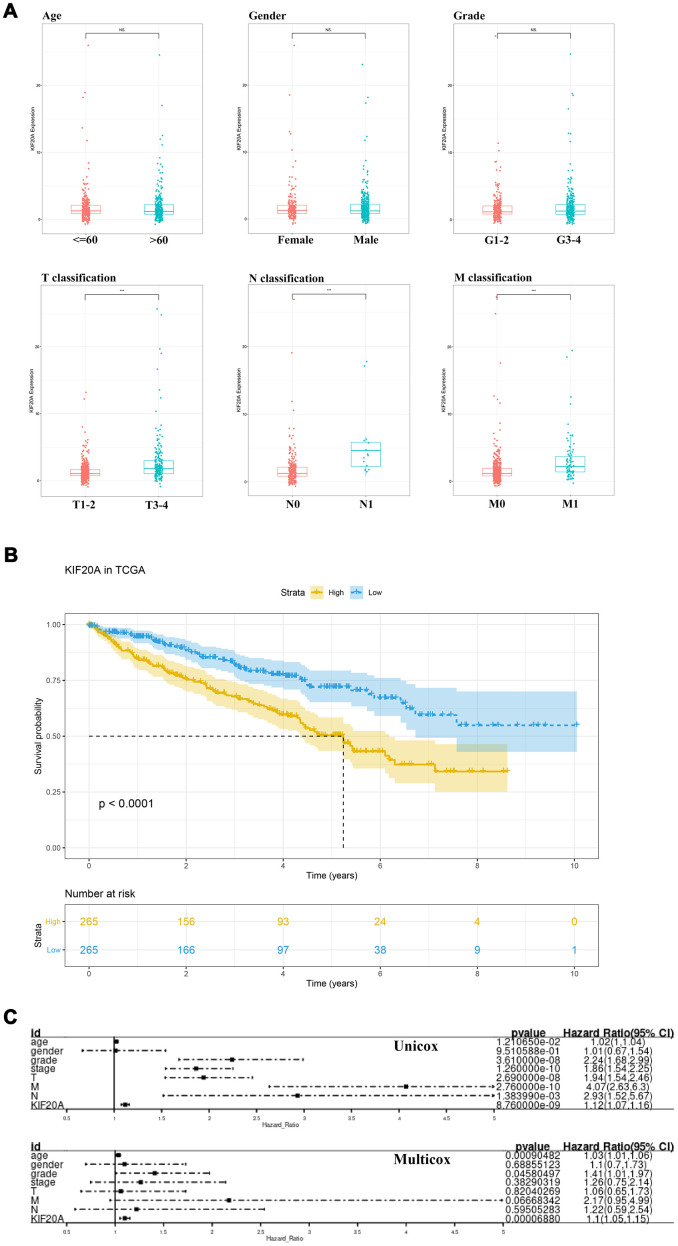
**The clinical correlation and prognosis analysis of KIF20A.** (**A**) The clinical correlation of KIF20A; (**B**) Kaplan-Meier curve of KIF20A expression in TCGA cohort; (**C**) Univariate and multivariate cox analysis of KIF20A and clinical variables.

The respective patient information, as deposited in the TCGA database, is shown in Supplementary Table 1. The association between each subset of clinical information and KIF20A levels was analyzed by R software according to the Wilcox test. We found that the levels of KIF20A expression continuously increased stepwise in each subgroup of T, N and M classification, suggesting that KIF20A may play a role in promoting a malignant phenotype (Figure 3A). The survival analysis revealed a shorter overall survival (OS) in patients with high KIF20 expression when compared to those with low KIF20 expression (P<0.0001) (Figure 3B). Results from single- and multi-factor analyses indicated that the negative effect of KIF20A towards patient survival was independent of other clinical features (Unicox, KIF20A, HR = 1.12, 95% Cl = 1.07-1.16, P < 0.001; Multicox, HR = 1.1, 95% Cl = 1.05-1.15, P < 0.001).

### Weighted gene co-expression network construction and key modules identification

**Figure 4:**
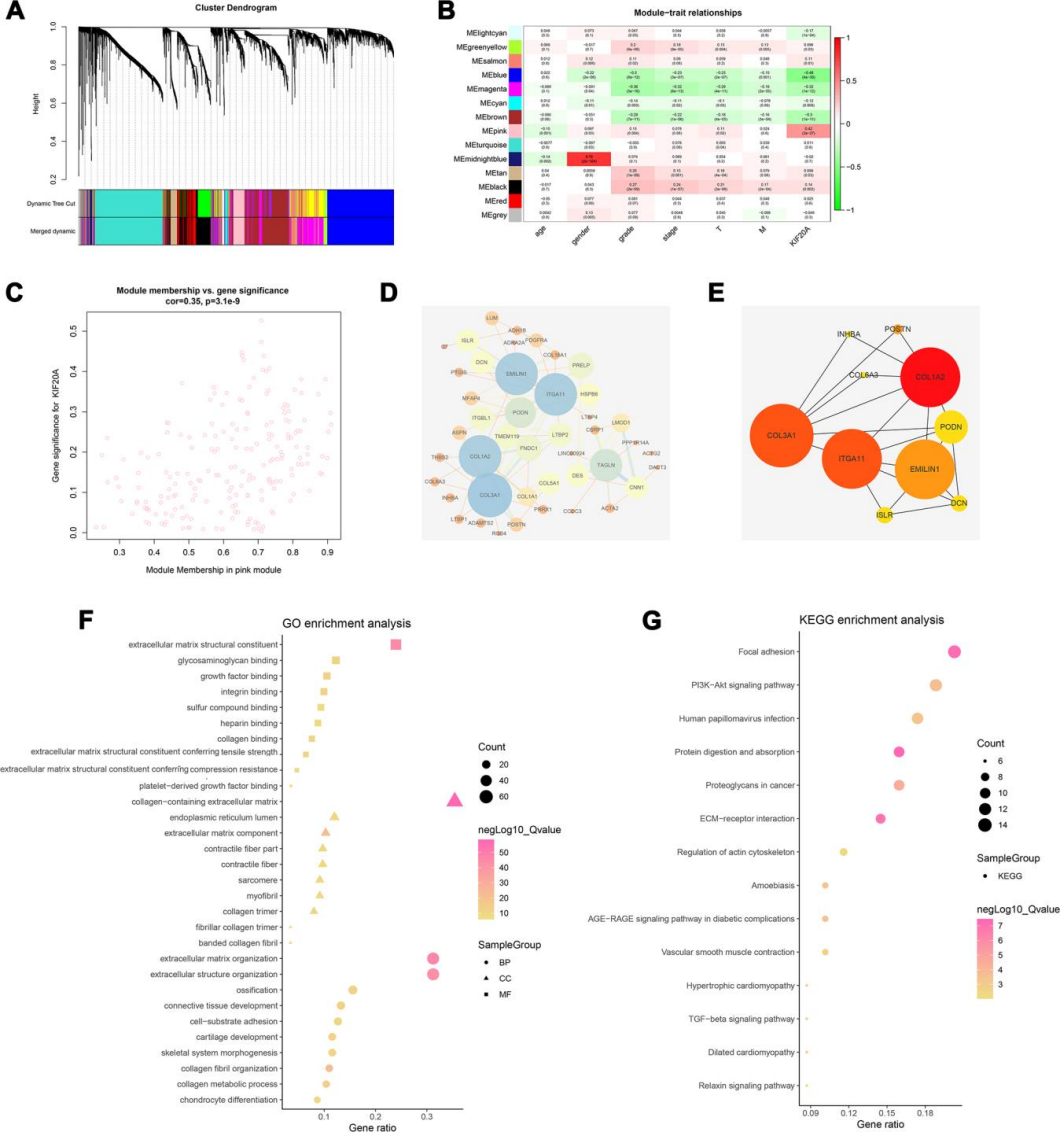
**Identification of modules associated with the KIF20A expression in the TCGA-KIRC dataset.** (**A**) The Cluster dendrogram of co-expression network modules were ordered by a hierarchical clustering of genes based on the 1-TOM matrix. Each module was assigned to different colors; (**B**) Module-trait relationships. Each row corresponds to a color module and column corresponds to a clinical trait. Each cell contains the corresponding correlation and P-value; (**C**) The pink module (the corresponding correlation and P-value); (**D**) Edges and nodes in pink module; (**E**) Top 10 nodes in PPI network; (**F**) GO analysis of module genes; (**G**) KEGG analysis of module genes. Abbreviations: PPI: Protein-protein interaction; GO: Gene Ontology; KEGG: Kyoto Encyclopedia of Genes and Genomes.

WGCNA analysis was conducted to identify gene modules associated with KIF20A expression in ccRCC. A total of 539 samples derived from ccRCC patients were clustered according to average linkage method and Pearson correlation analysis. Next, a network topology analysis of various soft-thresholding powers was performed to have relatively balanced scale independence and average connectivity of WGCNA. To make sure we developed a scale-free network, a power of β= 6 (scale-free R^2^) was selected as soft-thresholding parameter. After merging modules with dissimilarity of less than 25%, 14 distinct gene modules were identified (Figures 4A, 4B). Correlation analysis was further performed between the MEs of each gene module and KIF20A. Subsequently, a pink module (Cor = 0.42, P = 2e-27) was identified according to its high correlation with KIF20A expression (Figure 4C). Thereafter, a PPI network was constructed based on the genes present in the pink module. In this case, the top ten nodes were COL1A2, COL3A1, ITGA11, EMILIN1, PODN, DCN, ISLR, POSTN, COL6A3 and INHBA (Figure 4D, 4E).

### GO and KEGG analysis

Based on the GO analysis, “extracellular matrix”, “collagen”, and “cytokine binding” were the prominent terms in the cellular components (CC), molecular functions (MF), and biological processes (BP) sections (Figure 4F). Specifically, the result of GO analysis revealed that for BP, module genes were mainly enriched in “extracellular matrix organization”, “cell−substrate adhesion”, “collagen fibril organization” and “collagen metabolic process”. Changes in CC were markedly enriched in “collagen−containing extracellular matrix”, “endoplasmic reticulum lumen”, “extracellular matrix component”. Lastly, changes in MF were primarily enriched in “extracellular matrix structural constituent”, “glycosaminoglycan binding”, “growth factor binding” and “integrin-binding”. On the other hand, KEGG analyses showed that the gene modules were strikingly enriched in “Focal adhesion”, “PI3K−Akt signaling pathway”, “Proteoglycans in cancer” and “ECM−receptor interaction” (Figure 4G).

### Gene set enrichment analysis (GSEA)

**Figure 5:**
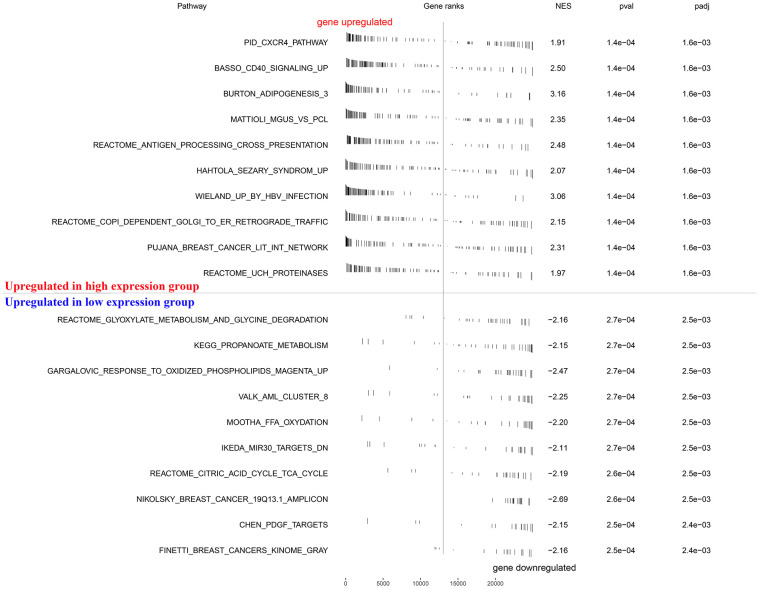
**GSEA enrichment analysis.**

To explore how the KIF20A gene was involved in ccRCC progression, we performed a GSEA based on the TCGA-derived ccRCC cohort. As shown in Figure 5, features including high expression phenotype, signaling pathways related to CXCR4 cascade, CD40 signaling-up, and burton adipogenesis were enriched (FDR < 0.25 and NOM P-value < 0.05).

### Immune analysis

**Figure 6:**
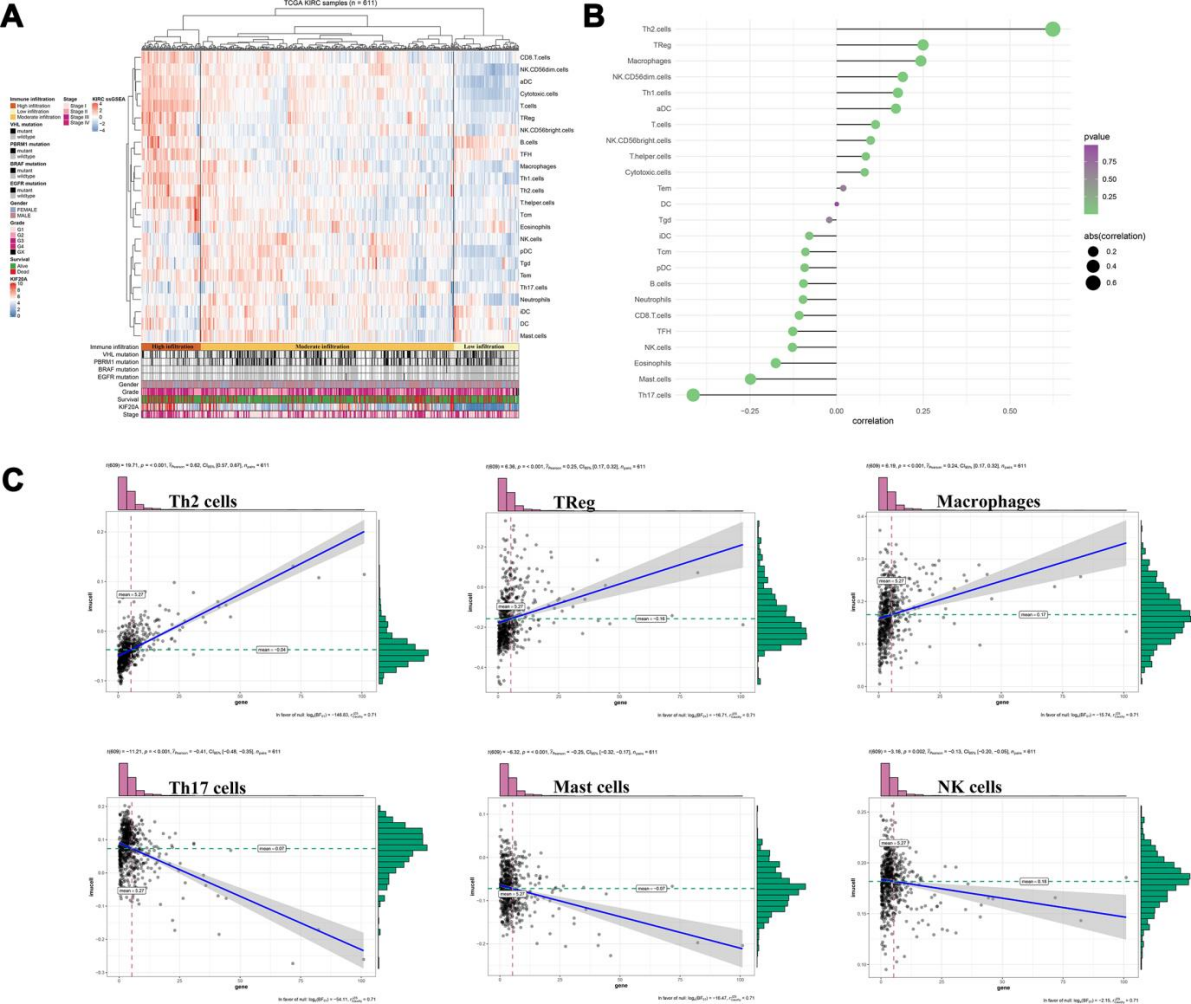
**Immune analysis of KIF20A.** (**A**) Immune infiltration in TCGA-KIRC samples; (**B**) The association between KIF20A and 24 immune cells; (**C**) The association between KIF20A and some immune cells. Abbreviations: TCGA, The Cancer Genome Atlas.

So far, our result suggests that samples with high KIF20A expression were likely associated with high immune infiltration in the tumour microenvironment (Figure 6A). Thus, further exploration of KIF20A in multiple immune cell populations showed that KIF20A had a strong positive correlation with Th2 cells, Treg cells and Macrophages, but a negative correlation with Th17 cells, mast cells and NK cells (Figure 6B, 6C).

### KIF20A is up-regulated in ccRCC tissue

**Figure 7:**
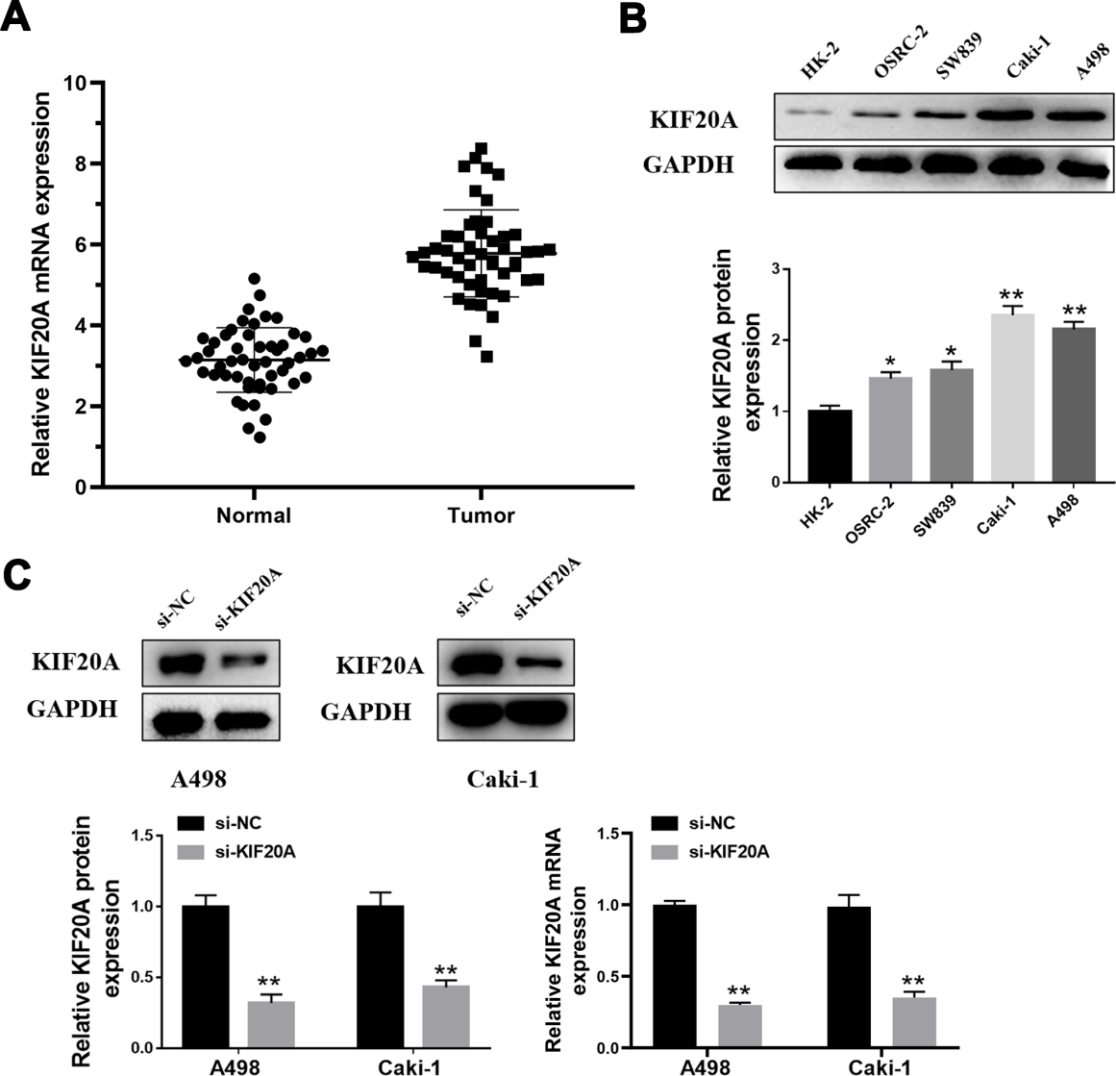
**KIF20A is up-regulated in ccRCC.** (**A**) Expression of KIF20A was frequently up-regulated in 50 ccRCC tumor samples compared with 50 adjacent healthy tissue by qPCR; (**B**) Western blotting of KIF20A expression in HK-2, OSRC-2, SW839, Caki-1 and A498 cell lines; (**C**) Western blotting and qPCR of indicated cells transfected with KIF20A-RNAi-vector, KIF20A-RNAi.

We performed qPCR analysis to further measure the KIF20A mRNA levels in a total of 50 tumors and respective adjacent normal tissues. As a result, a prominent increase on KIF20A mRNA levels was observed in tumor tissue (Figure 7A). Coincidentally, higher levels of KIF20A protein was also observed in renal cell carcinoma cell lines (OSRC-2, SW839, Caki-1 and A498), where A498 and Caki-1 presented the highest protein levels (Figure 7B). Hence, A498 and Caki-1 cell lines were selected for subsequent knockdown experiments. As such, western blotting and qPCR analysis revealed the successful knockdown of KIF20A in vitro upon transfection of small interfering RNAs (Figure 7C).

### KIF20 inhibits the apoptosis of renal cancer cells

**Figure 8:**
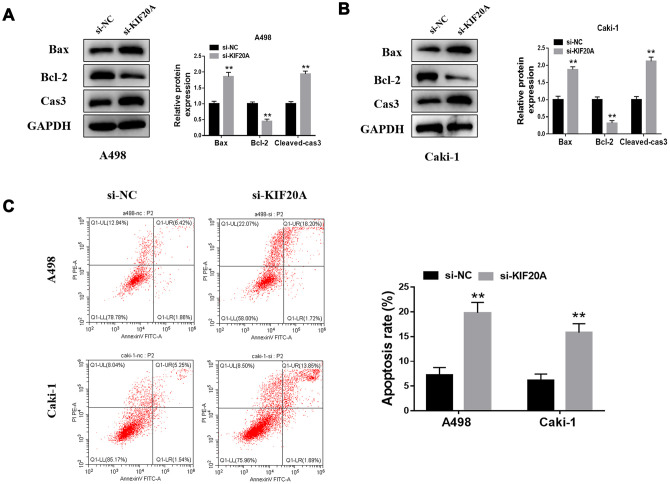
**KIF20A modulates the apoptosis of renal cancer cells.** (**A**, **B**) Detection of apoptosis related proteins by western blot in A498 and Caki-1 cell lines; (**C**) Cell apoptosis was detected by flow cytometry.

Western blotting analysis showed that KIF20A knockdown may significantly increase the protein level of Bax and cleaved-Cas3 and, contrarily, decrease Bcl-2 protein level (Figure 8A, 8B). Furthermore, flow cytometry results manifested a higher apoptosis rate in the si-KIF20A group when compared to control cells (Figure 8C).

### KIF20A promotes proliferation and invasion of renal cancer cells

**Figure 9:**
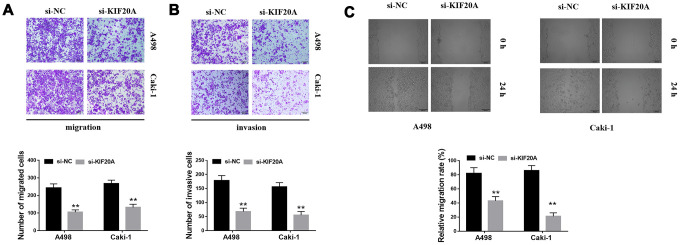
**KIF20A regulates the invasion of renal cancer cells.** (**A**, **B**) Downregulation of endogenous KIF20A reduced the number of invasion and migration cells in the transwell assay; (**C**) Wound-healing assay revealed that downregulation of endogenous KIF20A significantly reduced the migration rate.

**Figure 10:**
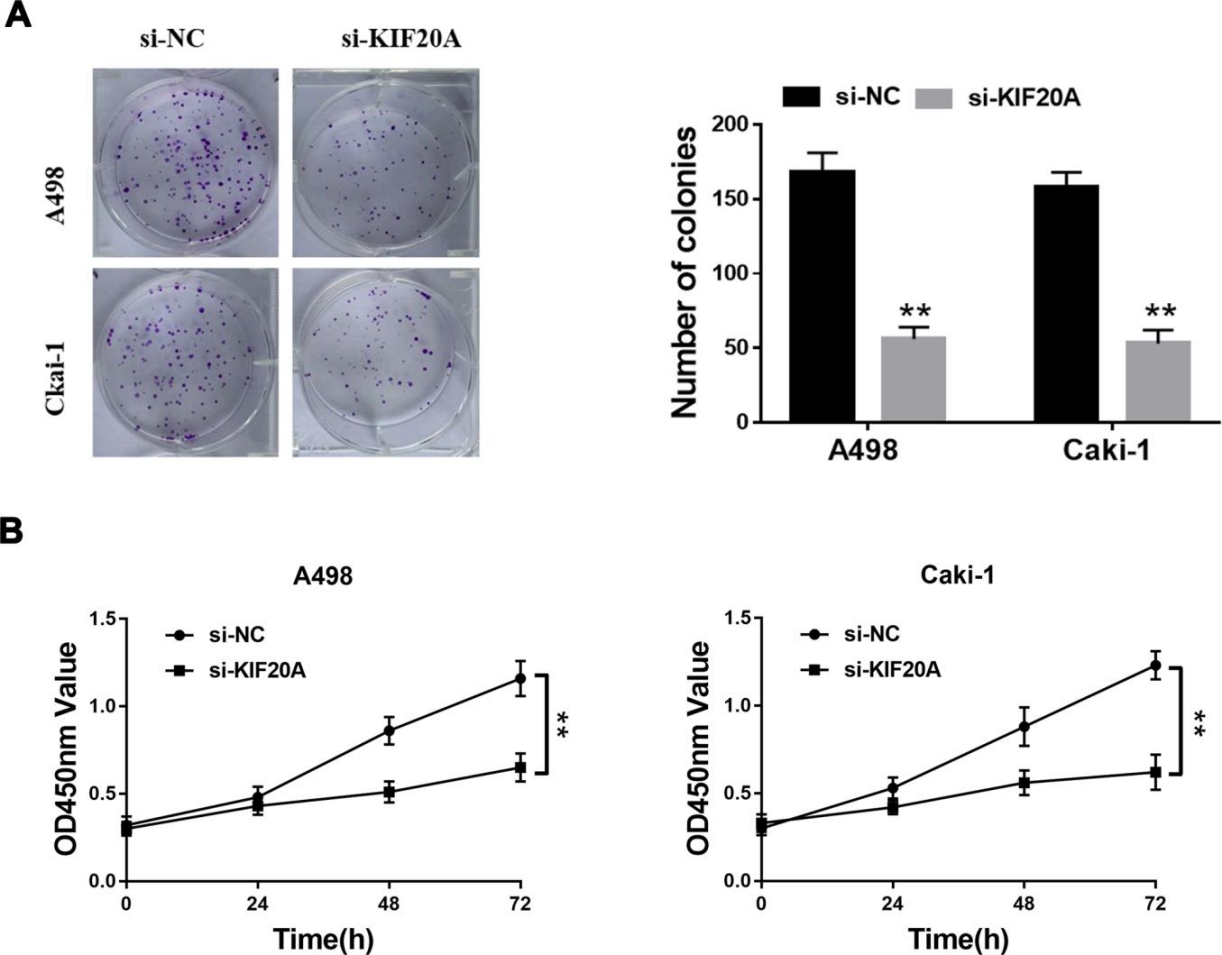
**KIF20A regulates the proliferation of renal cancer cells.** (**A**) Downregulation of endogenous KIF20A reduced the mean colony number in the colony formation assay; (**B**) MTT assays revealed that downregulation of endogenous KIF20A significantly reduced the cell viability.

Based on the results of transwell assays, a significant decrease on the content of migrating and invasive cells was detected in the si-KIF20 group (Figure 9A, 9B). This observation was further validated by wound-healing assays, in which a prominent wound healing area with lower migration rate was equally observed in the si-KIF20A group when compared with the control (Figure 9C). Knockdown of KIF20A expression can also significantly decrease the number of colonies based on a clonogenic assay (Figure 10A). Likewise, MTT assay result indicated that KIF20A knockdown is able to inhibit the proliferation of renal cancer cells (Figure 10B).

## DISCUSSION

The precise coordination of mitosis and cytokinesis can greatly influence the survival status of eukaryotes. As a critical molecule involved in the mitotic process, KIF20A has been previously reported to be overexpressed in multiple cancers, serving as a stimulus for their malignant phenotype [16, 17]. However, the expression pattern, molecular function and its correlation with clinical features remain unclear.

To the best of our knowledge, this is the first study which focuses on the role of KIF20A in ccRCC patients. In this work, we were able to confirme that KIF20A is overexpressed in renal cancer tissue and could also promote the proliferation and metastasis of related tumor cells. Of note, we have also identified KIF20A as an effective tumor and prognosis marker. Besides, KIF20A overexpression appears to be associated with high immune infiltration and specific immune cells. Altogether, our current data indicates that KIF20A may be a potential immunological and therapeutic target in ccRCC patients.

According to the expression profile obtained from multiple open access databases, KIF20A was proved overexpressed in ccRCC tissue. The AUC areas of ROC curves for predicting ccRCC were all > 0.9, inferring a robust prediction value for KIF20A during tumorigenesis. Clinical correlation analysis has demonstrated a positive association between KIF20A with T, N and M classification. These findings prompted us to explore the effect of KIF20A towards the malignant phenotype of cancer cells. Upon performing a series of in vitro experiments, we found that KIF20A is up-regulated in ccRCC tissue, and could promote the proliferation and invasion of renal cancer cells. Moreover, patients with high KIF20A expression appear to have a poorer prognosis when compared with the low KIF20 expression group from the TCGA cohort, thus indicating that KIF20A has a reliable prognostic value.

WGCNA analysis was further conducted to identify the gene modules most related to KIF20 expression. As a result, a pink module was selected. After GO and KEGG analyses, the gene modules were mainly in areas including extracellular matrix (ECM) and collagen. ECM, a macromolecular compartment mainly consisted of protein and polysaccharide, is capable of sustaining and preserving the normal epithelial structure. For instance, ECM can form a complex grid structure, supports and connects the tissue structure, regulate the tissue occurrence and cell activities [18]. In the context of cancer, ECM may play a vital role in the invasion and metastasis process of transformed cells [19]. Upon analysis of Affymetrix microarrays from 15 primary tumor tissues and matched metastases, Ho and colleagues have identified a number of ECM-related genes in molecular events that lead to visceral, bone and soft tissue metastases in ccRCC [20]. Additionally, Yamamoto and colleagues have verified that collagen 1, a main ECM component, can enhance cell invasion to further facilitate metastasis of renal cancer cells [21]. According to the large number of recent studies focusing on the impact of ECM in ccRCC, the detrimental effect of ECM on all progression steps of this malignancy has been undeniably proved [22], which is also consistent with our analytical results.

Considering the interconnection between KIF20A and the immune system, we further evaluated the effect of KIF20A in immune cell infiltration [14, 15]. Our results revealed that KIF20A is correlated with high immune infiltration of ccRCC tumor microenvironment. In this case, KIF20A was able to remarkably increase the numbers of Th2 cells, macrophages, and Tregs in the tumors, in contrast to the decrease on the numbers of Th17 cells, mast cells and NK cell. Based on a report from Senbabaoglu and colleagues, which explored the effects of tumor-infiltrating immune cells on disease prognosis, the increase of Th17 cells and CD8+ T/Treg ratio were both associated with improved survival in two cohorts [23]. Besides, Roumenina and colleagues have found that ccRCC cells may hijack macrophage-produced complement C1q, thereby promoting tumor growth [24]. The accumulation of NK cells in ccRCC microenvironment has also been considered associated with enhanced patient survival [25]. According to the retrospective analysis of 662 non-metastatic ccRCC samples, Fu and colleagues have suggested that patients with high mast cell infiltration might have a better OS, cancer-specific survival (CSS) and relapse-free survival (RFS) [26]. The evidence mentioned above may explain the poor prognosis of high KIF20A patients from the perspective of tumor immune microenvironment. These results indicate that KIF20A potentially have a reference value for the relief and immunotherapy in patients affected by ccRCC.

Nevertheless, our current research possesses certain limitations. Firstly, data series downloaded for further analysis were mainly originated from Western countries (only eight samples were from Asian), so the results of the study may not apply to patients in Asian countries. Secondly, subsequent studies are still needed to properly dissect the mechanism by which KIF20A promotes the progression of renal cancer. Thirdly, the amount of ccRCC data available in the public database is still limited, so the clinical pathology parameters used for our analysis are not comprehensive and may lead to potential errors/biases.

## CONCLUSIONS

Based on a series of bioinformatics analysis and in vitro experiment, we have presently shown that KIF20A is up-regulated in ccRCC tissues. Moreover, KIF20A expression appears to promote the proliferation and invasion of tumor cells. Hence, KIF20A potentially serve as a useful tumor and prognosis marker. Due to its interaction with immune cells (from the tumor microenvironment), KIF20A may in fact act as an underlying immunotherapy target in ccRCC.

## MATERIALS AND METHODS

### Data acquisition

Open data were retrieved from the TCGA database (https://portal.gdc.cancer.gov/; TCGA-KIRC), including the expression profile (Primary site: kidney, Program: TCGA, Project: TCGA-KIRC, Data Category: transcriptome profiling, Data Type: Gene Expression Quantification, Workflow Type: HTSeq – Count), clinical feature and survival information (Primary site: kidney, Program: TCGA, Project: TCGA-KIRC, Data Category: clinical, Data Type: Clinical Supplement, Data Format: bcr xml) of 539 ccRCC samples and 71 normal samples [27]. The ICGC database (https://icgc.org/; Last updated on: 31 May 2020) provided complete transcriptome information and clinical data of 91 tumor samples and 47 normal samples [28]. Chip data derived from ccRCC patients were downloaded from GSE40435 (Platform: GPL10558; 101 tumor samples and 101 adjacent no-tumor renal tissues), GSE36895 (Platform: GPL570; 29 tumor samples and 23 normal cortex tissues), GSE46699 (Platform: GPL570; 65 tumor samples and 65 normal normal tissues) and GSE53757 (Platform: GPL570; 72 tumor samples and 72 normal normal tissues) [29–31]. The “affy” package of R software was used to read the original CEL files from the GEO database. The KIF20A mRNA levels of normal renal tissues were retrieved from the Genotype-Tissue Expression (GTEx) Portal [32]. The names of samples originated from TCGA and GTEx databases were manually matched. The immunohistochemical images illustrating KIF20A levels in tumor and normal tissues were obtained from The Human Protein Atlas project (HPA) [33].

### Data processing and clinical correlation analysis

Gene expression profile data were pre-processed using R software. For this, the following variables were adjusted: (i) background correction, (ii) data normalization, (iii) removal of batch effects, (iv) combination of normal and tumor group data, (v) ID transformation gene symbols, and (vi) probing of supplemental missing values. Since the expression profile of KIF20A did not satisfy a normal distribution and variance homogeneity, Wilcox test was performed to compute the correlation between KIF20A expression and clinical features. The “survival” package in R was used to visualize the Kaplan-Meier curves in both high and low KIF20 groups. The “pROC” package was utilized to assess the predictive ability of KIF20A in tumorigenesis.

### Weighted correlation network analysis

To identify the significant mRNAs associated with KIF20A expression in ccRCC, we constructed a co-expression network using the WGCNA package. The goodSamplesGenes function was applied to check whether the differentially expressed mRNAs (DEmRNAs) of the data matrix meet the criteria and, moreover, to eliminate any unqualified data. The “pickSoftThreshold” function was used to calculate the value of β (a soft threshold power parameter) to ensure a scale-free network. Next, a tree diagram was designed according to hierarchical clustering and the correlation between module eigengenes (MEs) and clinical traits was measured for the screening of the MEs related to the ccRCC-specific KIF20A expression

### PPI network construction and functional enrichment analysis

To further understand the mechanism of protein interaction and cellular processing, we constructed a PPI network based on the co-expression network of the WGCNA module. For this, the edges with an interaction score of >0.3 were selected. Cytoscape_v3.8.0 was used to visualize the PPI network. Cytohubba, a plug-in of Cytoscape, was further utilized to analyze the top 10 nodes containing the highest amount of interactions. The package of “clusterProfiler” in R software was used for GO and KEGG enrichment analyses. P<0.05 served as a cut-off of statistical significance.

### Gene set enrichment analysis

GSEA was performed between high KIF20A and low KIF20A patients to study the biological characteristics of our model. For this, the following cut-off criteria were applied: (i) “collapse data set to gene symbols” was false; (ii) the number of marks = 1000; (iii) phenotype as a “permutation type”; (iv) the “enrichment statistic” was weighted; (v) FDR <0.25, and nominal P-value <0.05 were cut-off criteria. Signal to Noise metrics were used for gene ranking. The high KIF20A group was regarded as an experimental group, while the low KIF20A group was defined as a reference. The “c2.cp.kegg.v7.1.symbols.gmt” gene set database was selected for the enrichment analysis.

### Immune correlation analysis

The “ssGSEA” package was used to quantify the content of immune cells in respective TCGA samples. The most significant advantage of this evaluation was related to the high degree of freedom in the quantification process. Information about maker genes in 24 immune cells was obtained according to Bindea and colleagues [34]. The correlation coefficients of KIF20A and immune cells were calculated according to the Spearman method.

### Patients and cell lines

Patient tissues used for PCR analysis were obtained from the First Affiliated Hospital of Nanjing Medical University. This study was allowed by the Ethics Committee of the First Affiliated Hospital of Nanjing Medical University. All patients had approved for the use of clinical tissues for research purposes.

Normal renal epithelial cell lines (HK-2) and the human renal carcinoma cell lines (OSRC-2, SW839, Caki-1 and A498) were purchased from iCell (Shanghai, China).

### Quantitative PCR (qPCR)

Total RNA was isolated using Trizol (Invitrogen). PrimeScript RT Master Mix (Takara, JPN) was used for first-strand cDNA synthesis. For the analysis of KIF20A mRNA levels, qPCR was further performed using SYBR Green according to the manufacturer’s instructions (Applied Biosystems, USA). The primers presently used were: KIF20A, forward: 5-TGCTGTCCGATGACGATGTC-3; KIF20A, reverse: 5-AGGTTCTTGCGTACCACAGAC-3; GAPDH, forward: 5-AC CACAGTCCATGCCATCAC-3; GAPDH, reverse: 5-TCCACCACCCTG TTGCTGTA-3.

### Western blotting

Total proteins were extracted from human ccRCC tissue with Western and IP lysis buffer (Beyotime, P0013; Beijing). Protein concentration was measured using the BCA reagent kit (Pierce, 23227). Proteins were resolved by 8-12% SDS-PAGE and then transferred to polyvinylidene fluoride (PVDF) membranes. Membranes were further blocked with TBS/0.1% Tween-20 (TBST) containing 5% skimmed milk powder for 1hr at room temperature. Next, blocked membrane was incubated with respective primary antibodies against KIF20A, Bax, Bcl-2, Cleaved-Casp3 or GAPDH (1:300 or 1:2,000 dilution, AtaGenix, Wuhan) for 2hrs at room temperature. Thereafter, membrane was washed and incubated with a diluted secondary antibody [anti-rabbit or anti-mouse IgG (H+L) biotinylated antibody (CST, USA)] for additional 2hrs at room temperature.

### RNA interference studies

The knockdown of *KIF20A* expression in vitro was performed using small interfering RNAs (siRNA). For this, Caki-1 and A498 cells were transfected with control or KIF20A-specific siRNAs using Lipofectamine 3000 (Invitrogen). The target sequences used for siRNA against KIF20A were sense: 5-GGCCAGGUUUCUGCCAAAATT-3, antisense: 5-UU UUGGCAGAAACCUGGCCTT-3. Western blotting and qPCR were used to evaluate the efficiency of small RNA interference.

### Flow cytometric

Apoptosis was assessed by Annexin V/propidium iodide (PI) staining, followed by flow cytometry (BD Biosciences) analysis, according to the manufacturer’s instructions. Briefly, harvested cells were washed once with phosphate-buffered saline (PBS) and once with a 1x-binding buffer. Next, cells were incubated with Annexin V-FITC and PI for 15 mins at room temperature and then analyzed by flow cytometry. Data analysis was conducted using FlowJo (Tree Star Inc., Ashland, OR) software.

### MTT assay

Cells were seeded in triplicates into a 96-well plate at the concentration of 2 × 103 cells/well. At specific incubation times, cells were treated with 100 μl of 0.5 mg/ml sterile MTT for 4hrs (37° C, 5% CO2; 24h, 48h, 72h). Medium was later removed and 150 μl of dimethyl sulphoxide (DMSO) was added per well. Cell viability was determined using MTT reagent.

### Clonogenic assay

Cells were inoculated into 30-mm cell culture dishes, containing respective media with 10% FBS and then cultured for 14 days. Culture medium was changed every three days. Cells were later fixed with 15% formaldehyde for 15 min and stained with 0.1% crystal violet for 20min before counting the resulting colonies.

### Wound-healing assay

Cells were seeded into a six-well plate and cultured to 90% confluency. Cell gaps were scratched with a 10 μl tip and set as 0 hours. Wound healing was observed after 24 hours of cell growth. The migration rate was calculated as follows: Migration rate = (initial wound area-specific day wound surface area)/initial wound area × 100%.

### Transwell invasion assays

Assay was carried out using a 24-well transwell chamber. For this, 2.5 × 10^5^ cells were added to the upper chamber and kept in a serum-free medium. The bottom chamber was filled with 500 μl of culture medium containing 20% FBS. After 24 hours, cells that were able to move to the lower surface of the membrane were stained with 0.1% crystal violet and photographed accordingly.

### Statistical analysis

Statistical analyses were performed using R (v. 3.6.1), SPSS (v. 23), ImageJ and FlowJo software. All statistical tests were two-sided, and P-value <0.05 was considered statistically significant. All experiments were repeated at least three times.

## Supplementary Material

Supplementary Table 1
