# Supplementary material for: Upregulation of KIF20A promotes tumor proliferation and invasion in renal clear cell carcinoma and is associated with adverse clinical outcome
Source: Aging (Albany NY). 2020 Nov 24;12(24):25878–94. doi: 10.18632/aging.202153 (PMC7803492; doi:10.18632/aging.202153)
Supplement: Supplementary Table 1 [file aging-12-202153-s001.pdf]

Supplementary Table

Supplementary Table 1. Correlation between KIF20A expression and clinicopathologic characteristics of ccRCC patients.

Clinical characteristics	KIF20A expression	
	Low or none, no. cases (%)	High, no. cases (%)
Age (y)		
<=60	129 (24.3%)	135 (25.5%)
>60	136 (25.7%)	130 (24.5%)
Gender		
Male	151 (28.5%)	193 (36.4%)
Female	114 (21.5%)	72 (13.6%)
Grade		
G1	10 (1.9%)	4 (0.8%)
G2	141 (26.7%)	86 (16.2%)
G3	91 (17.1%)	115 (21.7%)
G4	17 (3.2%)	58 (10.9%)
GX (or unknow)	6 (1.1%)	0 (0%)
Clinical stage		
Stage I	166 (31.3%)	99 (18.7%)
Stage II	35 (6.6%)	22 (4.2%)
Stage III	44 (8.3%)	79 (14.9%)
Stage IV	20 (3.8%)	62 (11.7%)
Unknow	0 (0%)	3 (0.6%)
T classification		
T1	169 (31.9%)	102 (19.2%)
T2	40 (7.5%)	29 (5.5%)
T3	55 (10.4%)	124 (23.4%)
T4	1 (0.2%)	10 (1.9%)
N classification		
N0	116 (21.9%)	123 (23.2%)
N1	1 (0.2%)	15 (2.8%)
NX	148 (27.9%)	127 (24.0%)
M classification		
M0	220 (41.5%)	200 (37.7%)
M1	18 (3.4%)	60 (11.3%)
MX (or unknow)	27 (5.1%)	5 (0.9%)
